# Developmental Changes in the Corpus Callosum from Infancy to Early Adulthood: A Structural Magnetic Resonance Imaging Study

**DOI:** 10.1371/journal.pone.0118760

**Published:** 2015-03-19

**Authors:** Megumi M. Tanaka-Arakawa, Mie Matsui, Chiaki Tanaka, Akiko Uematsu, Satoshi Uda, Kayoko Miura, Tomoko Sakai, Kyo Noguchi

**Affiliations:** 1 Department of Psychology, Graduate School of Medicine and Pharmaceutical Sciences, University of Toyama, Toyama, Japan; 2 Department of Pediatrics, Graduate School of Medicine and Pharmaceutical Sciences, University of Toyama, Toyama, Japan; 3 Department of Radiology, Graduate School of Medicine and Pharmaceutical Sciences, University of Toyama, Toyama, Japan; 4 Department of Physiology, School of Medicine, Keio University, Tokyo, Japan; Institution of Automation, CAS, CHINA

## Abstract

Previous research has reported on the development trajectory of the corpus callosum morphology. However, there have been only a few studies that have included data on infants. The goal of the present study was to examine the morphology of the corpus callosum in healthy participants of both sexes, from infancy to early adulthood. We sought to characterize normal development of the corpus callosum and possible sex differences in development. We performed a morphometric magnetic resonance imaging (MRI) study of 114 healthy individuals, aged 1 month to 25 years old, measuring the size of the corpus callosum. The corpus callosum was segmented into seven subareas of the rostrum, genu, rostral body, anterior midbody, posterior midbody, isthmus and splenium. Locally weighted regression analysis (LOESS) indicated significant non-linear age-related changes regardless of sex, particularly during the first few years of life. After this increase, curve slopes gradually became flat during adolescence and adulthood in both sexes. Age of local maximum for each subarea of the corpus callosum differed across the sexes. Ratios of total corpus callosum and genu, posterior midbody, as well as splenium to the whole brain were significantly higher in females compared with males. The present results demonstrate that the developmental trajectory of the corpus callosum during early life in healthy individuals is non-linear and dynamic. This pattern resembles that found for the cerebral cortex, further suggesting that this period plays a very important role in neural and functional development. In addition, developmental trajectories and changes in growth do show some sex differences.

## Introduction

The corpus callosum, which consists of approximately 200 million fibers of varying diameters and degrees of myelination, is the largest inter-hemispheric commissure of the human brain [1]. This structure plays an important role in signal transmission across the left and right hemispheres. Studies of split-brain patients and those with agenesis of the corpus callosum have advanced our understanding of the neurological and functional connectivities between the two hemispheres [2,3], which are crucial to the brain’s ability to perform complex functions [2,4].

Each hemisphere has specialized functions [2]. The left hemisphere is typically involved in speech and language processing, whereas the right hemisphere specializes in visual-spatial processing and facial recognition [2]. Many callosal projections are homotopic. Namely, they connect equivalent regions between the two hemispheres. The fiber connections between corresponding hemispheric regions are topographically arranged across the callosum [5]. It has been suggested that such connections are modality-specific and the anterior callosal fibers interconnecting the frontal lobes transfer motor information, and posterior fibers connecting the parietal, temporal, and occipital lobes bilaterally are responsible for the integration of somatosensory (posterior midbody), auditory (isthmus), and visual (splenium) information [6]. Furthermore, previous studies have revealed a correlation between the development of white matter structure and the development of motor skills and cognitive functions [7,8]. These findings suggest that morphometric development of the corpus callosum is associated with the maturation of various human brain functions.

Previous studies have investigated the corpus callosum in terms of two main topics: Development and sex differences [9–12]. The development of the corpus callosum has been examined using post-mortem, light microscope, magnetic resonance imaging (MRI), cranial sonographic imaging, transvaginal sonography and diffusion tensor imaging (DTI). Inter-hemispheric fibers are formed at the genu at around the eighth week of gestation, with formation of the fibers extending in the rostro-caudal direction [13]. The structure of the corpus callosum is eventually formed at approximately 20 weeks gestation [13]. It should be noted that the corpus callosum develops together with related areas of the brain and continues to increase in volume after birth [13,14]. A DTI study of neonates has demonstrated that mean diffusivity (MD) of the genu decreased significantly, whereas fractional anisotropy (FA) increased significantly with gestational age, suggesting that nerve fibers become more mature and organized with advancing development. Conversely, the nerve fibers of the splenium were different from those of the genu, and MD and FA of the splenium did not change with gestational age. [15]. Furthermore, Pujol et al.[16] reported that the size of the corpus callosum increases up to the mid-twenties, with a more rapid growth rate in the early years and slower growth in subsequent years. We have previously described a “growth spurt” of the human cerebral cortex at around two years of age [17]. Research of foetuses using tansvaginal transducers has demonstrated that the width and thickness of the corpus callosum grow rapidly between 19 and 21 weeks of gestation [18]. The development of the corpus callosum does not appear to be linear. Myelination processes also affect morphometric development of the corpus callosum. Histological and MRI studies have verified that myelination of the corpus callosum begins at around 4 months of age and continues throughout adolescence [14]. Studies of children and adults suggest that myelination processes might affect the size of the corpus callosum. Giedd et al.[19] reported that size of the corpus callosum increases in a linear fashion with age.

Sex could be an important biological factor in the development of the corpus callosum, with the presence or absence of the Y—chromosome and the production of gonadal hormones contributing to differences between the sexes. DeLacoste-Utamsing and Holloway [20] initially reported that total corpus callosum area is larger in females than in males, which attracted considerable attention to the topic of sex differences in the corpus callosum. Since then, numerous researchers have investigated sex differences in corpus callosum morphology. Fetal testosterone hormone levels play a role in the asymmetric development of the corpus callosum, although no relationship was found between corpus callosum size and fetal testosterone levels [21]. However, results are inconsistent across previous studies. For example, some researchers have argued that the splenial area of the corpus callosum is significantly larger in females than males [10,22–24], while others report such a sex difference for the isthmus area [25,26]. Giedd et al.[11] reported that the genu area is significantly larger for males than females. These discrepancies could be due to age ranges of the participants studied, and it is important to take age into account when investigating corpus callosum changes [10].

Previous studies have shown structural changes in the corpus callosum throughout childhood and early adulthood [16,19] as well as sex differences in various corpus callosum subregions [9–11,22], and have also reported morphometric anomalies and sex differences of the corpus callosum in various neuropsychiatric disorders [27–30]. Although some previous studies have examined morphometric developmental trajectories of the corpus callosum [11, 12], other studies have focused on the analysis of the corpus callosum of infants [15, 31]. Moreover, a few studies have directly observed developmental characteristics by targeting a wide age range, including infants. This study examined the morphology of the corpus callosum in healthy participants of both sexes, aged 1 month to 25 years old. The well-accepted methodology for measuring postmortem brains developed by Witelson [25] has been previously used for the measurement of corpus callosal size from *in vivo* MRI recordings [11, 12, 19]. Witelson [25] devised an approach for defining the subareas of the corpus callosum according to their anatomical connectivity. The corpus callosum, because of the orientation and extensive myelination of its fibres, sharply contrasts with its neighbouring grey matter and the cerebrospinal fluid tissue. Therefore it is easily visualized on MRI [11, 12, 14, 19]. Moreover, all the components of the corpus callosum are present by 20 weeks of gestation [13, 14] and the corpus callosum has been visible on the MRI even in the first month of life. Segmentation method of subareas based on Witelson [25] was determined by the definite rate of the total length. We were able to measure the subareas of the corpus callosum in infants less than one year old.

## Materials and Methods

### Participants

The present study included 114 healthy Japanese subjects (61 males and 53 females) aged 1 month to 25 years old (mean ± s.d.: 105.7± 82.7 months), who were recruited as part of a cross-sectional study. The recruitment period lasted from October 1998 to July 2010. Participants younger than 18 years old were family members of university hospital staff, children of parents who were attending a community class on raising children, or children attending school in the Kureha region of Toyama city. Participants aged 18 years and older were recruited from the University of Toyama or were acquaintances of hospital staff. The age and sex distributions for the participants are shown in Table 1. There was no sex difference in age (in months) between males and females (males: 96.9 ± 86.4 months, females: 115.9 ± 77.8 months; *t* = -1.227, *df* = 112, *ns*). All participants were born full term (gestational age between 37 and 41 weeks) without any complicated perinatal courses. The mean participant heights and weights by years-based age categories were also shown in Table 1 and all were in the normal range referring mean Japanese heights and weights by results of the body survey in 2012 from Ministry of Health, Labour and Welfare Bureau, Health Division, lifestyle-related diseases Response Office. All participants were right-handed using the Japanese Hand Preference Questionnaire [32, 33], and were screened via an interview using questionnaires for perinatal, early developmental, educational, medical, neurological or psychiatric abnormalities. Parents of children younger than 18 years were also interviewed. Neurodevelopmental assessment was performed by well-trained clinical psychologists during the same period as the MRI scan, within one week after the scan. Nineteen children under 42 months of age were evaluated using the Bayley Scales of Infant Development, second edition (BSID-II). The mean mental development index was 97.1 (SD = 11.5), and the mean psychomotor developmental index (PDI) was 97.7 (SD = 10.5). Thirty children from 48 months to 16 years were evaluated using the Wechsler Pre-school and Primary Scale of Intelligence (WPPSI) or the Wechsler Intelligence Scale for Children, third edition (WISC-III). The mean IQ for this group was 109.6 (SD = 13.0). The Minnesota Multphasic Personality Inventory (MMPI) was administered to participants aged 18 years and older, and participants were excluded if any *T*-score exceeded 70. After the purpose and procedures of the study were fully explained, written informed consent was obtained from the participants, and/or the participants’ parents if the participants were below 18 years old. This study was reviewed and approved by the Research and Ethics Committees at the University of Toyama.

**Table 1:** Frequency, height and weight of Participants.

Age (Years)	Male			Female			Total
	N	height(cm) mean(SD)	weight(kg) mean(SD)	N	height(cm) mean(SD)	weight(kg) mean(SD)	N
0–1	14	65.1 (7.4)	7.1 (1.9)	5	62.0 (8.3)	7.0 (1.6)	19
1–2	5	77.5 (4.1)	10.3 (1.2)	5	76.7 (1.4)	10.7 (1.7)	10
2–4	7	90.2 (4.1)	13.5 (1.9)	5	92.6 (4.2)	14.1 (1.0)	12
4–6	4	106.7 (7.5)	17.1 (1.7)	3	108.3 (10.6)	19.8 (4.5)	7
6–8	4	118.3 (2.4)	22.8 (4.2)	2	117.3 (4.6)	20.1 (2.3)	6
8–10	3	127.7 (5.5)	27.0 (4.4)	11	132.2 (1.9)	27.0 (3.4)	14
10–12	4	141.5 (5.0)	40.0 (13.7)	3	135.5 (2.1)	33.7 (1.5)	7
12–14	4	167.0 (2.3)	50.5 (5.3)	4	165.0 (1.0)	43.3 (2.9)	8
14–16	4	169.0 (8.5)	60.8 (7.1)	5	159.0 (6.6)	50.0 (6.3)	9
16–18	6	168.3 (2.8)	63.0 (5.5)	4	160.0 (2.6)	48.7 (3.1)	10
18–20	2	163.5 (2.1)	59.3 (11.9)	2	163.0 (2.8)	54.3 (1.5)	4
20–25	4	173.0 (2.1)	72.3 (6.3)	4	162.0 (2.0)	56.3 (6.5)	8
Total	61			53			114

### Image Acquisition

T1-weighted axial images were obtained on a clinical scanner (Magnetom Vision; Siemens, Erlangen, Germany), using the fast low angle shot 3-dimensional gradient refocused sequence with the following parameters: Echo time (TE) = 6 ms, repetition time (TR) = 35 ms, flip angle = 35°, nex = 1, field of view = 256 mm, and matrix size = 256 x 256, slices thickness = 1.0 mm and 110–180 contiguous slices were obtained in each case. The number of slices were positively correlated with the whole brain volume, which was calculated based on the method described below (r = .62, p<.001). Total scan time was 15 minutes. Forty-seven participants below 6 years old were sedated with Triclofos sodium syrup (0.5–1.0 ml/kg) before the scans. This sedative is safe and routinely used in clinical situations.

### Image analysis

MR images selected for further analysis were encoded and stored on a computer without personal identifiers. Image processing was performed using ANALYZE 10.0 semiautomatic image analysis software (Mayo Clinic, Mayo Foundation, Rochester, MN, USA). First, DICOM data were converted to ANALYZE format (hdr and img). Next, noise was reduced using the Noise Reduction Tool (Susan) in Functional Magnetic Resonance Imaging of the Brain Software library (FSL: www.fmrib.ox.ac.uk/fsl/). The software ANALYZE allows the rater to view the image voxels in three mutually orthogonal planes simultaneously, any one of which may be stipulated by the rater. Brains were realigned in three dimensions to standardize for differences in head tilt during image acquisition. The tilt in the sagittal plane was corrected by aligning the anterior commissure—posterior commissure (AC–PC) plane, in the axial and coronal planes by aligning the longitudinal third ventricle and the interhemispheric fissure by reference to symmetry of the eyeballs and the optic nerves.

**Fig 1:**
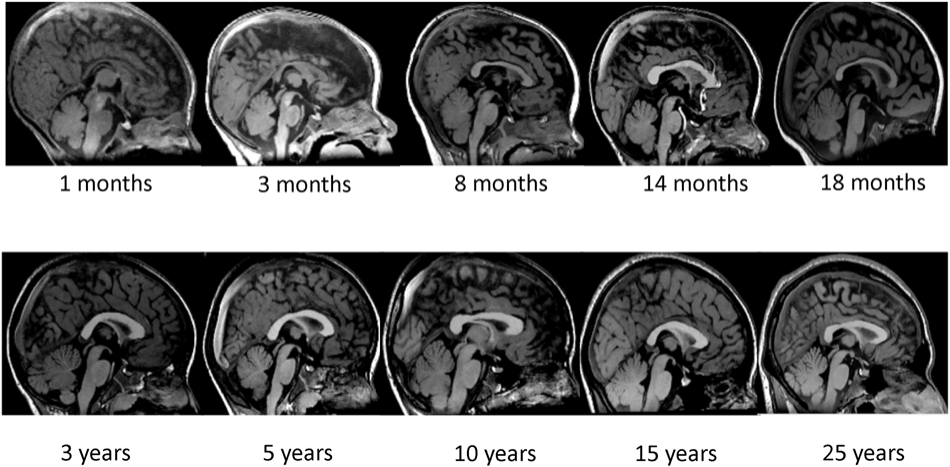
The samples of the corpus callosum on midsagittal MRI.

**Fig 2:**
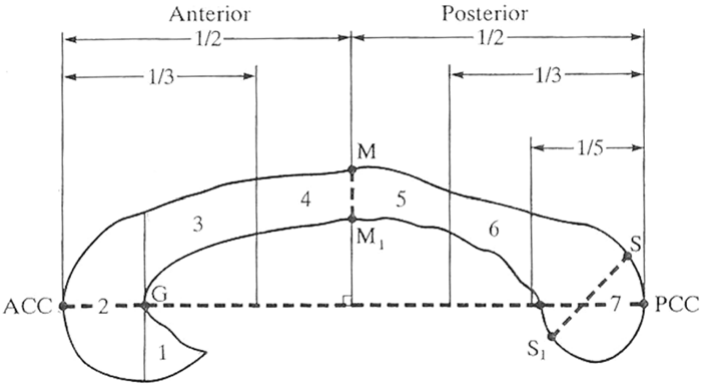
Regional subdivisions of the corpus callosum based on Witelson et al. (1989) and Venkatasubramanian et al. (2007). Subregions: (1) rostrum, (2) genu, (3) rostral body, (4) anterior midbody, (5) posterior midbody, (6) isthmus, (7) splenium.

The border of the corpus callosum was then traced in midsagittal orientation. Following Giedd et al. [11], a line was drawn to bisect the cerebral hemispheres from the axial 3D data set at the level of the anterior commissure and posterior commissure to accurately capture the midsagittal plane. From this line, a midsagittal image was reconstructed. The midsagittal orientation criterion was confirmed by the presence of the cerebral aqueduct, septum pellucidum, and the distinctness of the thalamus. The samples of the corpus callosum on midsagittal MRI were shown Fig. 1. The segmentation of the corpus callosum into seven subareas was performed using image processing software (ANALYZE 10.0); the corpus callosum was segmented into the rostrum, genu, rostral body, anterior midbody, posterior midbody, isthmus and splenium using the parcellation scheme described by Witelson [25] and Venkatasubramanian et al. [34] (Fig. 2).

Whole brain volumes were measured based on Matsuzawa et al. [17]. For the whole brain, all supratentorial slices were analyzed and the infratentorial cerebral-spinal fluid (CSF) and associated tissue were excluded by placing a boundary around the posterior fossa on each slice.

Twenty scans were randomly selected to examine inter- and intra-rater reliability (ten infants or children and ten adolescents or adults, male vs. female = 10:10), with inter-rater reliability being assessed across three raters (M.M.T-A., S. U. and K. M). Intraclass correlation (ICC) analysis for the seven subdivisions and the entire corpus callosum yielded an average value of 0.99 (infants/children: 0.98; adolescents/adults: 0.99; female: 0.98; male: 0.99). The intra-rater reliability was 0.98 (infants/children: 0.99; adolescents/adults: 0.97; female: 0.99; male: 0.97). The rater M.M.T.-A. then completed the analysis on the remaining scans, being blind to age and sex of subjects in MRI data.

### Statistical Analysis

Developmental patterns were analyzed using the nonparametric local smoothing model called locally weighted regression analysis (LOESS) [35] with R 2.15.0 (http://www.r-project.org/) software. The LOESS approach permits analysis of curve form using actual data. Using this technique, age-related volume changes could be delineated by applying the curve fitting suggested in previous human studies [36, 37] by using a relatively few data points, without enforcing a common parametric function on the data set, as is the case with linear polynomial models. The fit at a given age was made by using values in a neighbourhood that included a proportion, alpha, and for alpha < 1, the neighbourhood included a proportion, alpha, of the values. Data were fitted in four interactions with alpha = 0.75. A robust fitting procedure was used that guarded against deviant points distorting the smoothed points [38]. In addition, from the LOESS data, the differentiation rate (growth change rate) in each month was calculated and graphed: Growth change rate = (an approximate value of present month—an approximate value of past month)/(an approximate value present month) × 100.

To assess different stages of development, we defined four age groups: Infants (age ≤ 2 years), children (2 years < age ≤ 10 years), adolescents (10 years < age <18years) and adults (18 years ≤ age ≤ 25years), according to ages at peak brain volumes [17, 39, 40]. An analysis of variance (ANOVA) was conducted with the seven regions as repeated measures, and sex (male, female) and age group (infants, children, adolescents, adults) as between-group variables. An ANOVA was also conducted to confirm the effects of sex and age on total corpus callosum (CC) and whole brain. In addition, sex differences in the ratio of total CC and each sub-area to whole brain were examined using Mann-Whitney U test. Post hoc Tukey’s tests were employed to follow-up the significant main effects of interactions yielded by ANOVA. We defined statistical significance as *p* < 0.05. To prevent a possible Type-I error due to multiple tests, Bonferroni corrections for numbers of subarea of corpus callosum were applied for post hoc comparisons such that p-values less than 0.001 were used to define statistical significance.

## Results

### Developmental Curves and Growth Changes

**Fig 3:**
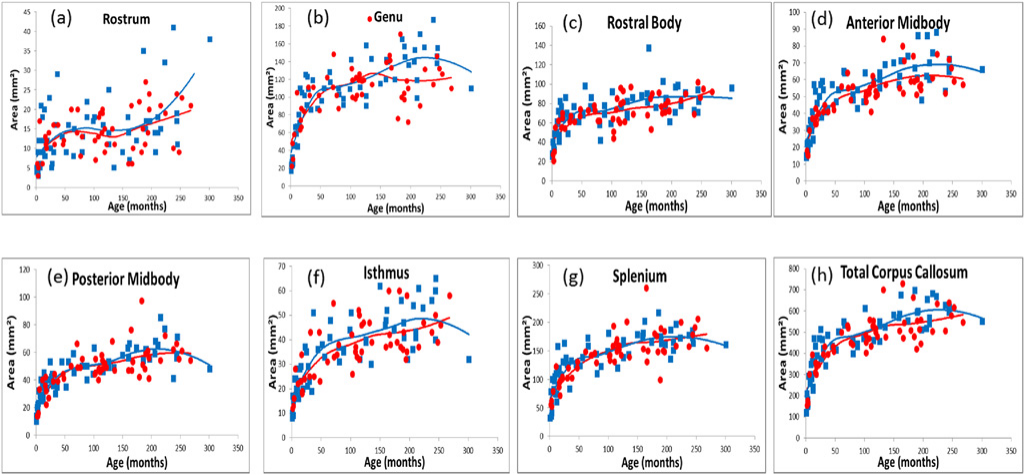
Scatter plots by monthly age and sex of absolute area of the each part of corpus callosum with local smoothing models (LOESS). (a); rostrum, (b) genu, (c) rostral body, (d) anterior midbody, (e) posterior midbody, (f) isthmus, (g) splenium, (h) total corpus callosum, blue square: male (n = 61), red circle: female (n = 53), blue line: males’ LOESS, red line: females’ LOESS.

Fig. 3 shows LOESS scatter plots for the corpus callosum. It can be seen that subareas, as well as the area of the overall corpus callosum all increased markedly from birth during the first 24 months (2 years), regardless of sex. After this initial increase, the curves of the slopes gradually became flat for both sexes.

**Fig 4:**
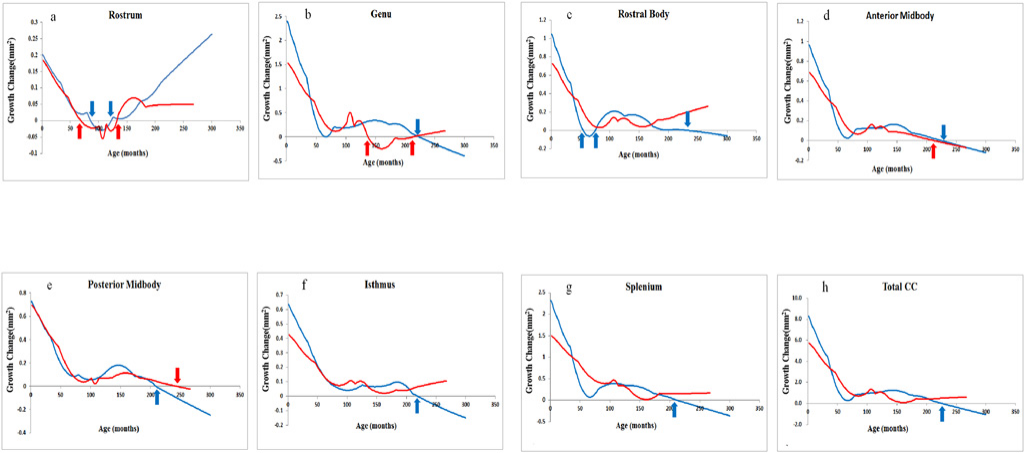
Rate of monthly growth change for (a) rostrum, (b) genu, (c) rostral body, (d) anterior midbody, (e) posterior midbody, (f) isthmus, (g) splenium, (h) total corpus callosum. Positive values indicate increasing size of the area. The point of intersection on the x-axis represents the age of local maximal area. Blue line: males’ LOESS (n = 61), red line: females’ LOESS (n = 53). The arrows show the local maximum for each region calculated using LOESS. For the rostrum, there were local maxima beyond 24 months (2 years) for both females and males, 69 (5.9 years) and 132 months (11 years) old for females and 86 (7.2 years) and 122 months (10.2 years) for males. For the genu, females had two local maxima at 137 (11.5 years) and 214 months (17.1 years), whereas males had only one local maximum at 223 months (18.7 years). For the rostral body, local maxima were observed only in males (55 (4.7 years), 75 (6.3 years), and 233 months (19.5 years)). For the anterior and posterior midbodies, local maxima were observed only once for both sexes (for females at 211 (17.7 years), males at 224 months (18.8 years); for females at 243 (20.3years), males at 209 months (17.5 years), respectively). For the isthmus, splenium and entire corpus callosum, single local maxima were observed only for males (219 (18.3 years), 212 (17.8 years) and 226 months (18.1 years), respectively).

Nonparametric local smoothing models revealed age (in months) to be significantly related to corpus callosum size. There also appeared to be developmental differences between the sexes. Fig. 4 shows changes in monthly growth rate for the entire corpus callosum, as well as each subarea. Ages when monthly growth rate change became zero (Fig. 4) correspond to the local maximums depicted in Fig. 3. The ages at which each subarea and the entire corpus callosum reached their local maximums differed across females and males. As indicated in Fig. 4, growth changes in the corpus callosum differed as a function of subregion and sex. Females showed more growth change variations in the anterior regions (rostrum, genu) of the corpus callosum, but relatively consistent growth change in the middle and posterior regions (rostral body, anterior midbody, posterior midbody, isthmus, splenium). In contrast, males showed similar growth changes for each region of the corpus callosum, with the exception of the rostrum. Rate of growth changes was dynamic, particularly for infants and children. However, at around 192 months (16 years) acceleration of growth changes started to flatten out in females, and the same happened in males at around 204 months (17 years).

### Comparisons of the regions among sex and age groups

Table 2 shows mean area of the corpus callosum by sex in infant, child, adolescent and adult groups. An ANOVA conducted for the seven CC regions showed main effects of age group (*F*(3, 106) = 75.34, *p* <. 0001) and regions (*F*(6, 636) = 1147.0, *p* <. 0001), but not a main effects of sex (*F*(1, 106) = 0.80, *p* = 0.37). Post hoc tests revealed all age comparisons except that between adolescents and adults were significant (adults = adolescents > children > infants, *p* <.001). There was a significant interaction of regions-by-age group (*F*(18, 636) = 27.10, *p* <. 0001), although there were no significant interactions of sex-by-age groups (*F*(1, 3) = 0.62, *p* = 0.60), regions-by-sex (*F*(6, 636) = 0.46, *p* = 0.84), or regions-by-age groups-by sex (*F*(18, 636) = 1.49, *p* = 0.09). Post hoc tests revealed that the genu and splenium (*p* <.001) of the infants group was significantly smaller than children, adolescents and adults groups. In addition, rostral body, anterior midbody and posterior midbody of the infants group was significantly smaller than that of adolescents (*p* <.001). Moreover the splenium of the children group was smaller than adolescents and adults groups (*p* <.001). There was no other significant difference among groups.

**Table 2:** Mean size of Corpus Callosum divided by sex and age groups.

	Infants(age ≤ 2 years)		Children(2 years < age ≤ 10 years)		Adolescents(10 years < age< 18 years)		Adults(18years ≤ age ≤25 years)		Age effect	
Region	Male(n = 19)	Female(n = 10)	Male(n = 18)	Female(n = 21)	Male(n = 17)	Female(n = 15)	Male(n = 7)	Female(n = 7)	*F*	*p*
Rostrum	10.2±6.2	9.7±4.5	13.8±6.0	14.0±4.3	15.3±7.1	14.4±4.3	24.7±12.03	18.1±6.1	3.68	0.014
Genu^1)^	55.1±28.8	63.7±25.7	108.6±15.4	111.6±15.1	127.9±26.4	126.5±29.2	151.9±24.5	121.3±18.3	4.75	0.003
Rostral Body^2)^	52.5±18.5	49.4±17.1	70.0±14.0	70.0±12.8	86.4±17.8	75.7±11.8	83.6±10.2	88.1±10.4	1.83	0.145
Anterior Midbody^2)^	31.3±13.2	30.5±10.4	52.5±8.3	51.0±6.9	65.7±10.2	61.7±10.6	69.6±10.6	63.3±8.3	9.2	0.001
Posterior Midbody^2)^	28.4±9.5	27.6±10.6	46.0±8.8	48.5±6.8	57.7±7.4	57.3±14.2	62.3±15.9	60.6±6.8	10.69	0.001
Isthmus	19.9±7.4	20.9±6.8	35.6±9.3	36.4±9.3	44.1±8.5	43.4±9.2	52.4±12.1	45.7±7.1	8.71	0.001
Splenium^1^ ^,^ ^3)^	84.8±35.9	82.3±22.7	135.4±23.4	141.6±21.5	169.5±20.3	162.5±37.8	175.4±12.5	177.9±21.6	6.94	0.001
Total CC^4^ ^)^	282.2±112.5	284.1±87.0	461.9±64.8	473.0±53.6	566.6±67.3	541.5±89.0	541.5±89.0	575.0±57.1	75.34	0.001

Values represent mean±s.d measured area (mm2). Post hoc comparisons following ANOVA with seven regions as repeated measures, sex and age as between-group variables.

1) Infants group was significantly smaller than children, adolescents and adults groups (p <.001).

2) Infants group was significantly smaller than adolescents group (p <.001).

3) Children group was smaller than adolescents and adults groups (p <.001).

For total CC, post hoc comparisons following ANOVA to test the effects of age and sex.

4) Infants<children<adolescents ≈ adults (p <.001).

An ANOVA for the whole brain showed main effects of age group (*F* (3, 106) = 61.36, *p* <. 0001; post hoc test, infants<children<adolescents ≈ adults) and sex (male>female, *F* (1, 106) = 7.30, *p* <. 01). There was no significant sex-by-age interaction (*F* (3, 106) = 0.84, *p* = 0.47). An ANOVA for total CC showed a main effect of age group (*F* (3, 106) = 75.34, *p* <. 0001; post hoc test, infants<children<adolescents ≈ adults), but not sex (*F* (1, 106) = 0.80, *p* = 0.37). There was no significant sex-by-age interaction (*F* (3, 106) = 0.62, *p* = 0.60). The mean ratio of total CC and each sub-area to the whole brain are shown in Table 3. Ratio of total CC to the whole brain was significantly higher in females compared with males (U test, p < 0.05). Also, each ratio of genu, posterior midbody, splenium was higher in females compared with males (U tests, p <0.05). There was no sex difference in the ratio of other sub-areas.

**Table 3:** Mean ratio of Corpus Callosum to the whole brain.

Region	Male (n = 61)	Female (n = 53)	sex effect	
			*Z*	*p*
Rostrum/w	0.013±0.007	0.013±0.005	0.65	0.511
Genu/w	0.093±0.026	0.103±0.022	2.24	0.025
Rostral Body/w	0.067±0.012	0.068±0.012	0.54	0.587
Anterior Midbody/w	0.048±0.010	0.050±0.009	0.85	0.396
Posterior Midbody/w	0.043±0.009	0.047±0.012	2.19	0.028
Isthmus/w	0.032±0.009	0.035±0.009	1.6	0.11
Splenium/w	0.124±0.025	0.135±0.026	1.96	0.05
Total CC/w	0.420±0.076	0.451±0.073	2.08	0.037

Values represent mean±s.d. rate of cc size divided by whole brain volume.

## Discussion

This study was the first to use MRI and LOESS to examine development of the corpus callosum in healthy individuals from infancy to early adulthood. The present results provided evidence that females and males differ in terms of developmental trajectories for this brain structure, with significant age-related changes discernable from infancy to early adulthood.

The present study demonstrated two principal results. First, the developmental trajectory of the corpus callosum appears to be non-linear. This result is consistent with the findings of a previous study [11]. The present data show that each area of the corpus callosum increases markedly in area, particularly in the first few months from birth and regardless of sex. This cerebral cortex follows a similar developmental trajectory. The peak of the growth spurt for the cerebral cortex occurs at around two years of age [17]. Garela et al [41] measured distances of different landmarks on the CC in 622 children ranging from 1 day to 15 years of age and reported rapid growth until three years of age followed by slower growth. Our results are not inconsistent with their findings. Their method is useful for clinical practice although they did not directly assess the size of the CC.

Second, the ages at which each subarea of the corpus callosum reached their local maximums differed between the sexes. As indicated in Fig. 3, corpus callosum development is dynamic and shows at least two local maxima. After attaining maximum developmental rate, growth changes started to flatten out at around 192 months (16 years) for females and 204 months (17 years) for males. This deceleration of growth might correspond to the age at which the myelination process tends to subside. De Lacoste et al. [5] reported that nerves fiber development differs across the corpus callosum subareas, and that the corpus callosum develops together with related areas of the brain. According to studies of both monkeys [42] and humans [43], the corpus callosum shows specific patterns of regional variability in axon density, size, and degree of myelination.

Additionally, there was no significant sex difference in the absolute size of the corpus callosum. However, the CC ratios to whole brain volume in the genu, posterior midbody, splenium sections and total CC were higher in females than in males. These results regarding sex difference are consistent with Jankle et al [44], who reported that females exhibited larger CC ratios than males, but that there was no significant difference in the absolute size of CC. Sex differences in the anterior and posterior sections were reported in previous studies [10,11, 22–24,43,45]. The studies suggest that sex differences in the anterior and posterior sections may be due to differences in parameters of the fibers, such as axon number, size or myelination processes. Rakic and Yakovlev [13] reported that the myelination process occurs even after birth, continuing through adolescence.

Although the present study provides a fresh and interesting set of observations, there are some limitations. First, this study analyzed only the size of the corpus callosum. Others studies have reported sex differences in shape and callosal thickness [46,47], which may influence development and sex differences in the corpus callosum. Second, all of our participants were right-handed. Habib et al.[48] reported effects of handedness and sex on the morphology of the corpus callosum (the corpus callosum was significantly larger in inconsistent right-handers than in consistent right-handers). Further research is needed to study the effects of dominant hand. Finally, MRI permits morphometric examination of the brain structures *in vivo*. Nevertheless, this method cannot be used to examine fiber composition or myelination processes. Methods such as diffusion tensor imaging (DTI) would be required to examine these aspects of corpus callosum development.

In summary, the healthy developmental trajectory of the corpus callosum during early life is non-linear and dynamic. This result is similar with our research of the developmental trajectory of the frontal lobe [39], denoting that this period plays a very important role in neural and functional development. This study did not find significant sex differences in overall size of the corpus callosum, but developmental trajectories and timing of growth changes did differ between sexes and ratios of total corpus callosum and genu, posterior midbody, and splenium to whole brain were significantly higher in females compared with males.

### Data Availability

The authors confirm that all data underlying the findings are fully available without restriction. All relevant data are within the paper.
