# Correction: Developmental Changes in the Corpus Callosum from Infancy to Early Adulthood: A Structural Magnetic Resonance Imaging Study

**DOI:** 10.1371/journal.pone.0133090

**Published:** 2015-07-13

**Authors:** The PLOS ONE Staff

There are errors in the Funding section. The correct funding information is as follows: This study was supported by a Grant-in-Aid for Scientific Research (B) 20330141, 26285155, Grand-in-Aid for Exploratory Research 26590143 and Grant-in-Aid for Scientific Research on Innovative Areas 26118707 from the Japan Society for the Promotion of Science (JSPS) and JSPS KAKENHI, Grand-in-Aid for JSPS Fellows 242666. The funders had no role in study design, data collection and analysis, decision to publish, or preparation of the manuscript.
